# Transdermal Fentanyl Patch Effectiveness in Postoperative Pain Management in Orthopedic Patients: Literature Review

**DOI:** 10.3390/jcm13247646

**Published:** 2024-12-16

**Authors:** Andrei Niculae, Ionel Alexandru Checherita, Ileana Peride, Mirela Tiglis, Razvan Ene, Tiberiu Paul Neagu, Dragos Ene

**Affiliations:** 1 Clinical Department No. 3, “Carol Davila” University of Medicine and Pharmacy, 020021 Bucharest, Romania; niculaeandrei@yahoo.com; 2 Department of Nephrology, County Emergency Hospital Ilfov, 022104 Bucharest, Romania; al.checherita@gmail.com; 3 Department of Anesthesia and Intensive Care, Emergency Clinical Hospital of Bucharest, 014461 Bucharest, Romania; mirelatiglis@gmail.com; 4 Clinical Department No. 14, “Carol Davila” University of Medicine and Pharmacy, 020021 Bucharest, Romania; 5 Clinical Department No. 11, “Carol Davila” University of Medicine and Pharmacy, 050474 Bucharest, Romania; 6 Clinical Department No. 10, “Carol Davila” University of Medicine and Pharmacy, 050474 Bucharest, Romania

**Keywords:** fentanyl patch, transdermal administration, postoperative pain, orthopedics, multimodal analgesia

## Abstract

Controlling pain after major orthopedic surgery may be challenging, and it is related to delayed recovery, the development of chronic pain, and analgesic dependence. It is well known that effective postoperative pain control can reduce hospital stays by ensuring a more rapid rehabilitation, thereby decreasing the overall costs. Despite the development of analgesics, the use of opioids and their derivates remains the cornerstone of treatment for patients with acute moderate-to-severe pain in association with general or regional anesthesia. To reduce the risk of side effects and opioid addiction, considering the alarming epidemiological reports in relation to opioid abuse, combined analgesic methods are used, in addition to lower dosages or different forms of administration, such as transdermal administration. Fentanyl transdermal patches appear to be effective in controlling postoperative pain as part of multimodal analgesic regimens in knee and hip surgery, shoulder arthroplasty, traumatic fractures, and one-day surgery; this treatment has fewer associated side effects and can be safely used even in patients with renal impairment. It is also recommended for postoperative pain management in combination with a femoral–sciatic nerve block during foot and ankle surgery.

## 1. Introduction

Considering the aging population, bone fractures represent a worldwide major public health issue and are an economic burden [1,2]. The increased incidence of osteoporosis in the general population, characterized by reduced bone mass and abnormal bone architecture, results in increased bone fragility, a high risk of fractures, and joint damage [3]. The Global Burden of Disease Study from 2019 showed that there were about 178 million cases of new fractures worldwide and 455 million cases of acute or long-term symptoms secondary to a fracture [2]. Over the years, studies have shown that severe postoperative pain is a widespread issue, being associated with decreased quality of life and satisfaction, delayed rehabilitation and ambulation, increased mortality and morbidity (especially pulmonary, cardiac, and gastrointestinal complications, sleep perturbances, negative psychological effects), the development of chronic pain, and increased costs for healthcare systems [4,5,6,7]. Orthopedic procedures are known to be responsible for the highest pain scores on the first postoperative day, along with other traumatic procedures and obstetric surgery, especially if it is an emergency procedure [8,9,10]. In addition to sleep disturbances, anxiety and episodes of dyspnea are reported following total knee or hip replacement (TKA, THA—primary surgery or revision) because of acute pain persistence or the development of chronic pain [11]. Even three months after surgery, pain recall was reported in the elderly following orthopedic surgery [12]. In an observational study including 2638 patients undergoing total joint arthroplasty, the prevalence of severe chronic pain was about 10% of the studied population [13].

Therefore, immediate aggressive analgesic management should be implemented after major surgeries to prevent the development of the negative effects of moderate-to-severe uncontrolled pain, and the role of anesthesiologist using new techniques and pharmacological adjuvants for optimum analgesia is crucial in the perioperative period to improve short- and long-term outcomes [6,14] Various methods can be adopted to control postoperative pain, including local and regional anesthesia techniques (spinal/epidural anesthesia, various regional blocks like femoral block, sciatic block, axillary/interscalene/supraclavicular/infraclavicular brachial plexus block, popliteal block, wrist block, ankle block), nonopioid analgesics (nonsteroidal anti-inflammatory drugs—NSAIDS, acetaminophen, uncompetitive N-methyl-d-aspartate (NMDA)-receptor antagonist ketamine, the selective norepinephrine and serotonin-reuptake inhibitors—SNRIs), and opioid analgesics, as part of multimodal analgesia regimens [7,15,16,17].

SNRIs (duloxetine, venlafaxine), used as adjuvants for perioperative pain control, appear to have a small number of side effects and are associated with opioid consumption reduction and reduced risk of acute and chronic postoperative pain development, increasing overall patient satisfaction [18,19]. Gabapentinoids (gabapentin, pregabalin), in association with a multimodal postoperative analgesic regimen, lead to proper pain control and increased patient satisfaction, especially for those at risk for suboptimal pain control (preoperative opioid use, anxiety, depression, comorbidities) [20,21]. Another adjuvant in anesthesia is magnesium sulfate (MgSO_4_), which is associated with reduced opioid and overall analgesic requirements, as well as prolonged anesthesia duration and reduced incidence of postoperative nausea and vomiting (PONV and shivering) [22,23]. Aside from the anti-arrhythmic effect, intravenous lidocaine may be used to control postoperative acute pain as part of individualized analgesia regimens, decreasing the dosages of associated analgesics and hospital length of stay [24,25]. Yang et al. published a study about the role of intranasal vasopressin in controlling postoperative pain after various orthopedic surgeries. The authors discovered that it decreases the visual analog scale (VAS) scores 2–4 weeks after the intervention with no influence on blood pressure, heart rate, body temperature, and respiratory rate [26]. The DEX-2-TKA trial (dexamethasone twice for pain after TKA) showed that two doses of 24 mg of dexamethasone, as part of a multimodal regimen along with local infiltration of analgesics, ibuprofen, and paracetamol, leads to reduced postoperative pain and morphine consumption [27]. Finally, the multimodal approach to pain control after orthopedic surgery should include not only pharmacological strategies but also cognitive and physical elements to improve the patient’s outcome [28].

Opioid analgesics are often required in the postoperative period as part of multimodal analgesic therapy in many types of major orthopedic surgery (especially in total hip/knee replacement and major fractures). Current evidence is against the preoperative use of opioids as a single analgesic. These types of analgesics can be administered by different routes: parenteral, neuraxial, oral, transdermal, intranasal, transmucosal, and rectal. When using the transdermal route, the skin must be intact and hair-free to maintain a constant drug release [29,30,31,32].

Due to the development of all the non-intravenous formulations, fentanyl is considered to be the most important opioid analgesic. Passive transdermal fentanyl patches (TFPs) are frequently prescribed whenever strong analgesia is required, being an important part of individualized pain regimens [33,34]. As for transmucosal and transdermal formulations of fentanyl, over the years, they have proven to have a high efficacy for cancer pain and emergencies, as well as in pediatric patients [35,36]. Proper pain control after orthopedic surgery increases patient comfort and promotes early mobilization and recovery [37]. Compared to patient-controlled analgesia (PCA) devices, transdermal formulations improve mobility and allow for the early intervention of physical therapists, which is of paramount importance after orthopedic surgery, reducing the overall length of hospital stay and costs [38].

All patients receiving analgesics, including opioids, should be carefully monitored during treatment, especially for mental and respiratory disturbances (i.e., signs of hypoventilation), the development of nausea and vomiting, and the onset of ileus (i.e., constipation). Special consideration should be taken for elderly patients undergoing major orthopedic surgery due to the presence of various comorbidities and subsequent polypharmacy, frailty, and altered cognitive status, and for patients with chronic renal failure, considering the altered pharmacokinetics and pharmacodynamics [29,39,40].

In a time of controversy regarding opioid use based on all the addiction problems, the scope of this narrative review is to raise awareness about the impact of transdermal fentanyl patches in multimodal analgesia to manage moderate-to-severe postoperative pain which frequently accompanies orthopedic elective or emergency procedures, and, consequently, their role in improving patients’ quality of life and postoperative rehabilitation.

## 2. The Effects of Fentanyl and the Main Opioid-Induced Disturbances

Fentanyl, a low-molecular-weight and short-acting synthetic opioid, is 50–100 times more potent than morphine, according to Paul Janssen, due to its selective action on μ-opioid receptors (MORs). Table 1 presents the main physicochemical properties of fentanyl, which allow it to be suitable for transdermal administration [41,42,43,44,45,46,47,48,49].

Opioids are commonly used in clinical practice as part of general or regional anesthesia for their sedative and analgesic effects, being an important element of the algorithm for treating acute and, in selective cases, chronic pain [50,51]. In addition to the well-known side effects of opioid consumption (nausea, vomiting, constipation, respiratory depression), new effects are being studied that occur as consequences of hemostasis-disrupting mechanisms, like opioid-induced endocrinopathies, immunomodulation (especially in the cancer population), imbalance of the oxidative–antioxidative status, carcinogenesis promotion, and mental cognitive disturbances [52,53,54,55,56,57].

The opioid-induced endocrine function changes induce osteoporosis and osteopenia, reduce sexual function and libido, and promote infertility. These are the results of the effects on the hypothalamic–pituitary–gonadal axis, decreasing the secretion of gonadotropin-releasing hormone (GnRH) by binding to hypothalamic opioid receptors [52,53,58]. Additionally, opioid-induced adrenal insufficiency is another problem, secondary to hypothalamus–pituitary–adrenal axis suppression (up to 29% of chronic users) [52,53]. The effect on this system is not well understood, but it appears that chronic opioid consumption reduces the production of corticotropin-releasing hormone (CRH), which subsequently decreases the production of adrenocorticotropic hormone (ACTH), but the ability of the pituitary gland to respond to CRH is also impaired, decreasing the production of cortisol and dehydroepiandrosterone (DHEA) [59,60].

Opioid immunomodulation appears to be related to the effect on MORs and nonclassical naloxone-insensitive receptors (NORs) [61]. In the perioperative period, moderate-to-high doses of fentanyl decrease the level of proinflammatory cytokines (IL-1, IL-6) in comparison to low-dose administration. It also induces an inhibition of natural killer (NK) cell cytotoxicity, especially in the first 24 h after surgery, in high doses [62,63].

## 3. Transdermal Fentanyl Patch

Fentanyl can be administered transdermally due to its small molecular structure and high lipophilicity, allowing it to be rapidly transported between the plasma and the sites of effect in the central nervous system with a wide distribution in the body and a transfer half-life of about 4.7–6.6 min [53,54,55,56].

**Figure 1:**
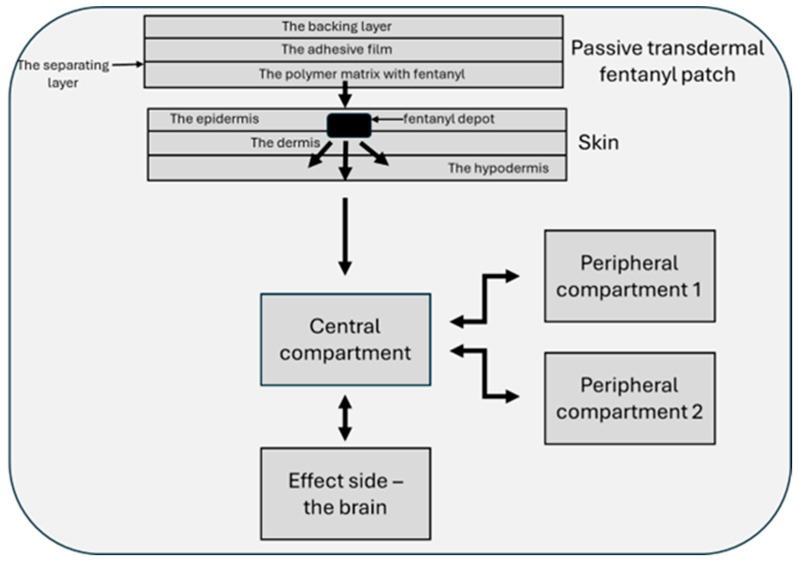
Model of transdermal fentanyl delivery [56,57,61,62,63,64,65,66].

Passive transdermal patches release fentanyl at a constant rate through the patient’s skin (Figure 1), with pharmacokinetic properties similar to the intravenously administered molecule [56,57,61,62,63,64,65]. Unlike other transdermal drugs, the percutaneous absorption of fentanyl is uniform regardless of the anatomical region (i.e., chest, thighs, abdomen) but may vary between individuals [64]. Bioavailability is higher because it does not have a first-pass metabolism, the delivery being the concentration gradient that develops between the transdermal patch and the skin, depending on the drug concentration in the matrix and the area of the skin where it is applied. This type of administration is based on a silicone matrix technique, where fentanyl is dissolved in an inert polymer matrix, which is responsible for drug delivery. The applied drug will traverse the epidermis due to its lipid solubility, and, at the dermal–epidermal junction, the absorbed fentanyl forms a depot and then slowly dissolves in the dermis layer where the large capillary beds are responsible for rapid absorption into the systemic circulation. Compared with transdermal systems using reservoirs, the passive forms exclude the risk of incidentally dumping the entire dose over a short period [47,61,65,66].

Typically, these patches deliver 12.5, 25, 50, 75, or 100 μg/h over from 18 to 72 h, depending on the local skin temperature (when at a body temperature of 40 °C, such as in cases of fever, the plasma concentration may increase by 33%), the state of skin hydration, underlying skin diseases, the patient’s age (decreased absorption in elderly), ethnic differences, the use of heating devices, and the presence of cancer [57,67,68]. It reaches a plateau concentration in 8–14 h after patch application and persists for a few hours after removal [69]. A recent study concluded that the drug concentration reached from 3 to 6 h after the application was sufficient to control pain [70].

TFPs are used in acute pain as part of a multimodal analgesic regimen or to treat chronic pain, especially in cancer patients [29,70]. Current trends recommend the use of transdermal fentanyl patches in association with other analgesics for one-day surgery, including orthopedic procedures, especially for patients at high risk of developing chronic pain (i.e., women, young people, obese individuals, patients with sleep and mood disorders, smokers) and those at low risk of opioid side effects [71]. In addition, Kwon et al. showed that a TFP placed 14 h before surgery not only has an anti-inflammatory effect by reducing the postoperative increase in interleukin-6 (IL-6) but also provides an analgesic response similar to the continuous infusion of fentanyl [72].

The use of passive transdermal fentanyl patches reduces the severity of side effects and the risk of overdose and abuse [73]. Studies have shown that the main side effects of fentanyl transdermal patches are pruritus, itching, edema, skin discoloration, erythema, papules, and dizziness [61,69,74].

## 4. Postoperative Pain Control After Orthopedic Procedures

Several studies have demonstrated the efficacy of transdermal fentanyl patches to control postoperative pain after gynecological or abdominal surgery [75]. It is also considered to be proper to control postoperative pain in dentistry interventions, like third molar surgery and cases of dry socket [76,77]. A TFP is also a good adjuvant for paravertebral block (PVB) in controlling postoperative pain after breast cancer surgery, as it is associated with reduced opioid consumption and minor side effects [78].

Considering the surgery time interval, the application of a TFP from 2 to 3 h before the surgical incision appears to be effective and has the same analgesic effect as morphine in orthopedic, abdominal, and thoracic procedures [58,79,80]. Current evidence is against the use of a TFP as a single agent in the treatment of acute postoperative pain. Also, it is not recommended to prescribe opioids before surgery [72].

Postoperative pain after total knee replacement was analyzed by Hall et al. in a randomized controlled trial. The authors concluded that a transdermal regime based on fentanyl ensures proper pain control comparable to standard patient-controlled analgesia (PCA), as it also has a safe profile with low side effects. It also provides a proper way to transition from hospital to home in the first week after surgery [81].

A study by Franklin et al., including 6364 patients with unilateral total knee arthroplasty (TKA), emphasized that 24% of patients received opioids before the procedure, 14% of which continued to use them at 12 months after surgery, and only 3% of those who did not receive it before TKA were still consuming at one-year follow up [82].

Abrisham et al. studied the effectiveness of a TFP after knee surgery in 40 patients undergoing general anesthesia and concluded that it reduces the use of morphine in the first 72 h after surgery, ensures effective pain control, and is not associated with additional side effects [83].

Minville et al. compared the use of a TFP (placed 14 h before surgery) and patient-controlled analgesia (PCA) for total knee arthroplasty; TFP, being effective 48 h after surgery, was found to reduce the pain scores (using the visual analog scale—VAS) and the level of morphine use. They emphasize the fact that transdermal patches are easy to use due to their increased skin adherence and that they decrease the need for an intravenous line and subsequent infections, are cheaper than pumps, and require no human programming, resulting in a decreased risk of errors [84].

In a previous study, both methods showed similar results, but a TFP was placed 2 h before surgery [85]. Caplan et al. used the patch before starting surgery and considered TFPs to be safe and effective in patients undergoing orthopedic surgery [86].

In knee surgery (especially total knee arthroplasty), TFP is considered safe; this is related to the use of a low morphine dose, increased patient comfort, and reduced hospitalization, favoring rapid ambulation due to lower pain scores both at rest and during movements, and, therefore, improving the rehabilitation and the risk of deep vein thrombosis [87]. It has been reported that pain scores can be reduced from 24 to 48 h after surgery, and muscle strength increases on the seventh day after the procedure [87,88].

Forefoot surgery (i.e., hallux valgus, hallux fracture, hallux rigidus, etc.) can be responsible for severe postoperative pain of up to 3 days. Merivirta et al. showed that postoperative TFP (12 µg/h) does not influence pain scores or opioid consumption compared to a placebo group [89].

A transdermal fentanyl patch was used by Vandeputte et al. as part of an enhanced recovery after surgery protocol to control postoperative pain after hallux valgus surgery. It was utilized in combination with nerve blocks, and the results showed a reduction in postoperative pain scores, the minimal use of strong opioids, and patient satisfaction at a high level. Only a small proportion of patients presented nausea [90].

Jokar et al. compared the effectiveness of TFPs and morphine in patients with leg bone fracture surgeries. It appears that TFPs, compared to morphine, have an increased analgesic effect, particularly for patients with symptoms like nausea and vomiting, dysphagia, opioid resistance, and those who are unable to take their medication [10].

For shoulder surgery, a study compared the use of TFPs and bupivacaine injection for postoperative pain control after arthroplasty. The authors concluded that fentanyl patches were safe and effective in controlling pain, reducing the episodes of breakthrough pain with minimal side effects [91].

Ebrahimzadeh et al. reported that, in a group of 281 patients undergoing orthopedic surgery, the results of using intravenous morphine as part of the patient-controlled analgesia (PCA) or TFP were similar in terms of safety and efficacy in controlling postoperative pain. However, TFPs are easier to use, have a lower risk of abuse, and are more cost-effective in Iranian hospitals, especially in trauma centers [62].

In a prospective study including 60 patients, compared with bolus or continuous epidural fentanyl administration, the role of TFPs in controlling postoperative pain after lower limb orthopedic surgeries appears to be inferior in terms of analgesia, resulting in better VAS scores, patient satisfaction, and reduced side effects [92]. In addition, they are also inferior to the injection of diluted anesthetic into the sciatic nerve for controlling postoperative pain after foot and ankle surgery [93]. Nevertheless, Song et al. showed that fentanyl patches applied after the procedure are useful to supplement postoperative analgesia and, consequently, for a femoral–sciatic nerve block in foot and ankle surgery [94]. Jokar et al. compared the effectiveness of TFP and morphine in patients with leg bone fracture surgeries. It appears that TFP, compared to morphine, has an increased analgesic effect, particularly for patients with symptoms like nausea and vomiting, dysphagia, opioid resistance, and those who are unable to take their medication [10].

When prescribing opioids for perioperative pain control, especially after major orthopedic surgery, the predictive elements for prolonged postoperative narcotic use should be thoroughly analyzed. Various studies, reviews, and meta-analyses on this matter have been conducted in recent years [95]. A systemic review by Lavoie-Gagne et al., including 458.993 patients, showed that the main risk factors for prolonged postoperative opioid use are a body mass index (BMI) of ≥40 kg/m, prior substance abuse, chronic pain conditions (chronic back pain, migraines, fibromyalgia), and psychiatric comorbidities [96]. After ankle surgery, one of the most painful orthopedic interventions, the main risk factors appear to be active tobacco use, alcohol abuse, and the presence of chronic comorbidities (diabetes, depression, low back pain) [97,98]. For total hip and knee arthroplasty, preoperative opioid use, age ≤ 65 years, high doses of opioids at discharge, post-traumatic stress disorder, benzodiazepine use, migraines, and fibromyalgia presence, are risk factors for prolonged narcotic use [99,100]. As for emergency orthopedic procedures, the presence of preoperative pain and anxiety, patient expectations regarding postoperative pain, and the use of general anesthesia are associated with increased pain scores and increased usage of opioids [9].

## 5. Special Considerations, Contraindications, and Precautions

### 5.1. Older Population

Older patients (with fall fractures or osteoarthritis) often require urgent or elective orthopedic surgery, total hip arthroplasty being the common elective procedure [101,102]. Another issue of importance for elderly individuals is represented by the underreported pain, “silent sufferers”, which, in most cases, occurs in relation to dementia or other cognitive perturbances [103]. Considering the rate of growth of the aging population [104], the need to manage postoperative pain after major surgery is increasing in the elderly due to changes in pharmacodynamics and pharmacokinetics [105]. In this group of patients, regardless of the method of administration, opioid consumption has many side effects, especially urinary retention, ileus, constipation, and delirium, which lead to prolonged hospitalization [106,107,108,109].

Current evidence suggests a reduction in opioid doses in older adults [109]. Elderly patients are often more sensitive to opioid agents, which increases the risk of side effects, especially respiratory depression [37]. A study published by Dagenais-Beaulé et al., which included 250 patients over 65 years who underwent elective or urgent orthopedic surgery for various fractures, found age-related changes in the use of opioid dosing among those undergoing elective surgery. The authors concluded that patients older than 80 years required lower doses of opioids compared to patients between 65 and 75 years old and that side effects such as cognitive deterioration, associated delirium, hypoventilation, and constipation may occur more often in this subgroup [110]. Bilek et al. also showed that, for older patients’ rehabilitation after surgery, the dosages of opioids should be considerably reduced [111].

### 5.2. Patients with Chronic Kidney Disease

The prevalence of chronic kidney disease (CKD) in the general population is about 13.1%, and >50% of these adults are older than 70 years [112]. Considering the lack of active metabolites, fentanyl appears to be safe for use in patients with renal failure. For severe pain, using fentanyl patches of 25 µg at 72 h may be considered [113]. Nevertheless, fentanyl is not recommended in hemodialyzed patients [114]. Some analyses have shown that the dose should be reduced in the presence of uremia because the clearance of the drug may be prolonged [88,108]. In the presence of renal impairment, the drugs with the safest pharmacological profile are alfentanil, fentanyl, buprenorphine, ketamine, remifentanil, and sufentanil [115,116].

### 5.3. Paediatric Patients

The use of TFPs in pediatric patients < 12 years old or in patients weighing < 50 kg is not recommended [69].

### 5.4. Opioid-Related Adverse Effects

In patients with chronic obstructive pulmonary disease (COPD), asthma, cor pulmonale, impaired respiratory status, cachexia, and elderly patients, fentanyl metabolism deteriorates, and respiratory depression can occur rapidly [117].

An analysis of the opioid-related adverse drug events (ORADEs) showed that these represent an important economic burden; a multimodal pain management approach over an opioid monotherapy decreases the risks (respiratory depression, nausea, vomiting, constipation, urinary retention, depression), and the related costs, with an incidence among surgical patients of about 1.8–13.6%. These patients are at risk of readmission, have longer hospital stays, and have higher overall hospitalization costs [118,119,120]. All ORADEs have a huge economic impact due to increased hospital costs and prolonged hospitalization [121].

A study published by Denke et al., including 27.942 surgical patients, showed that 90% were exposed at least once to opioids after surgery, and 0.3% presented serious ORADEs needing naloxone administration [122]. Some of the main general risk factors associated with ORADE development are male sex, age over 65 years, history of opioid use, COPD, cardiac dysrhythmias, gastrointestinal issues (diverticulitis, ulcerative colitis, regional enteritis), and psychiatric disorder [120]. After major orthopedic surgery, almost 2.5% of patients discharged with an opioid prescription develop ORADEs. The main independent risk factors are age over 80 years old, female sex, nervous system injury, musculoskeletal disorders, and chronic comorbidities (heart failure, respiratory illness, kidney disease, dementia, and anxiety disorders) [123].

The burden of perioperative opioid overuse is alarming and should raise awareness among all surgical and non-surgical medical specialties dealing with pain control, especially considering the level of worldwide opioid addiction and the subsequent deaths, as well as the distressing fact that 10–14% of patients undergoing orthopedic surgery end up taking opioids from 90 days to one year after discharge [124,125,126].

## 6. Conclusions

A transdermal fentanyl patch delivers the drug into circulation in a stable and reliable manner, reducing the risk of toxicity and side effects. Although widely used to manage chronic pain, especially that related to cancer, current guidelines and practices favor its use as an adjuvant or as part of a multimodal regimen in controlling postoperative pain in order to increase patient comfort and satisfaction, to reduce overall opioid use, and to promote early ambulation after orthopedic surgery. There are studies with good results for total knee and hip arthroplasty, shoulder arthroplasty, fractures, and foot and ankle surgery. In addition, TFPs are easier to use, have a lower risk of abuse, and can be successfully applied in CKD patients, although not in those undergoing chronic hemodialysis. Considering some of the main issues for our healthcare systems, like the aging population and the high incidence of chronic kidney disease, as well as opioid abuse, further studies regarding TFP use in controlling acute pain in patients undergoing various orthopedic interventions may help to improve overall outcomes.

## Figures and Tables

**Table 1:** The main physicochemical properties of fentanyl.

Parameter	Value
Molecular weight	336.471 g/mol
pKa	7.89–8.6 (at 15–47.5 °C)
Aqueous solubility (µg mL^−1^)	1:30–100
Octanol: water partition coefficient (lipophilicity)	~717
Skin flux (µg cm^−2^ h^−1^)	1
Protein binding	70% (42% at albumin)
t_1/2,ke0_	4.7–6.6 min

pKa = acid dissociation constant; t_1/2,ke0_ = transfer half-life between plasma and effect site.

## Data Availability

The database is available upon reasonable request to the corresponding authors.
